# Whole-genome analysis of *haemophilus influenzae* invasive strains isolated from Campinas state University hospital. An epidemiological approach 2012 - 2019 and ancestor strains

**DOI:** 10.1016/j.bjid.2021.101667

**Published:** 2021-12-24

**Authors:** Rafaella Fabiana Carneiro Pereira, João Paulo de Oliveira Guarnieri, Carlos Fernando Macedo da Silva, Bruno Gaia Bernardes, Marcelo Lancellotti

**Affiliations:** Biotechnology Laboratory, LABIOTEC, Faculty of Pharmaceutical Sciences (FCF), Campinas State University UNICAM, São Paulo, SP, Brazil

**Keywords:** Whole-genome, *Haemophilus influenzae*, Sequencing, Invasive disease

## Abstract

Thirteen *Haemophylus influenzae* invasive strains isolated from patients at Clinical Hospital of State University of Campinas, from May 2013 through August 2019, was submitted to Illumina genome sequencing HiSeq platform. Further *in silico* analysis of serogroup and Multi Locus Sequence Typing (MLST) from whole DNA sequencing had demonstrated the actual clonal distribution in the Campinas Metropolitan region. Thus, results showed the existence of a new ST *Haemophilus influenzae* found in the Brazilian territory and an increase of strains belonging to serogroup a (three strains also belonging to ST23). In conclusion, we observed an increase of non-typable *H. influenzae* (NTHi) and a strain involved in invasive diseases in the Campinas – São Paulo region after frequent detection of those serotypes and genotypes in other Brazilian regions.

## Introduction

*Haemophilus influenzae* is an important pathogen involved in several invasive diseases that might progress to meningitis, septicemia and death. Also, *Haemophilus influenzae* is known as pleomorfic Gram negative cocobacilus classified in six immunological encapsulated strains (a-f) and non-typable *H. influenzae* – NTHi. The *H. influenzae* type b (Hib) is the most invasive type commonly associated with meningitis and other upper respiratory tract infections in children and adults.^1^^,^^2^ The NTHi strains are associated with moderate diseases of the upper respiratory tract and otitis media in children and pneumonia in adults with cystic fibrosis.^3^^,^^4^ Along with the introduction of Hib conjugate vaccine, epidemiology of *H. influenzae* has changed in recent years. NTHi and other serotypes of *H. influenzae* has become more prevalent than Hib around the world. Outbreaks at the Clinical Hospital of University of Campinas, São Paulo State – Brazil have shown an increase of invasive strains after vaccination. The use of Illumina platform for bacterial whole-genome construction set up by our group for characterization of Brazilian Pupuric fever *Haemophilus* strains (Pereira *et al.* 2019^5^) was used for this purpose. This work aimed to use the Illumina sequencing method for draft-genome to characterize genome structure and *in silico* virulence factors of 13 invasive strains isolated from blood and cerebrospinal fluid. In addition, an approach about the virulence mechanisms and vaccine escape will be explored in this work.

## Material and methods

### Bacterial strains

Thirteen *H. influenzae* strains were isolated between May 2013 and August 2019 from patients in the Clinical Hospital of State University of Campinas (HC-UNICAMP). All strains were isolated from blood cultures except one Hi2015-6 isolated from cerebrospinal fluid. Hi38 and Hi45 strains had been characterized by Lancellotti *et al.* 2008^6^ and also isolates from the same hospital in 1998. The bacterial strains were grown in chocolate agar plates or BHI supplement with NAD (4 µg/ mL) and hemin (10 µg/ mL) (Kilian, 1976^7^) and incubated at 37°C with 5% CO_2_.^6^

### Whole-genome sequencing, assembly, and annotation

Genomic DNA was extracted as described and adapted by Cury *et al.* 2014.^8^ The DNA quality analysis and quantification were performed with NanoDrop (NanoDrop® 2000 - Thermo Scientific®). Libraries were prepared with the Nextera XT DNA library preparation kit (Illumina, CA, USA) and sequenced using the Illumina HiSeq 2500 platform (100-bp single-end reads) at the Genomics section of the Life Sciences Core Facility (LaCTAD, Campinas, São Paulo, Brazil). All libraries were multiplexed on one sequencing run. Quality of reads files were evaluated with FastQC v.0.11.7 (Babraham Bioinformatics, Cambridge, UK). Sequencing reads were trimmed, assembled, and annotated in through PATRIC pipeline v.3.5.43 (https://patricbrc.org/).^9^ Reads were trimmed by quality (Quality Phred score > 20) and size (> 20 pb) and Illumina adapters sequences were removed using the FastqUtils tool with Trim Galore v. 0.6.1 and Cutadapt v. 2.2. Assembly and annotation were performed using the tool Genome Comprehensive Analysis with SPAdes v. 3.10.0^10^ with default parameters and RAST tool kit (RASTtk),^11^ respectively.

### Capsular operon analysis and MLST determination

Capsular genes were identified using a BLASTn search with Hicap software^12^^,^^13^ and Geneious Prime® 2020.1.1 (https://www.geneious.com). For the Multi Locus Sequence Typing (MLST) determination, genes sequences for the housekeeping genes *adk, atpG, adk, atpG, frdB, fucK, mdh, pgi*, and *recA* were analyzed at *Haemophilus influenzae* MLST website (https://pubmlst.org/hinfluenzae/) sited at the University of Oxford^14^ for allele and sequence type (ST) assignment.

### Virulence genes and antimicrobial resistance genotypes

Virulence factors and acquired resistance genes were assessed using the Virulence Factors Database (VFDB)^15^ and ResFinder v. 2.1,^16^ respectively.

## Results

The sequencing of strains using the HiSeq2500 platform generated a total of 214,376,882 reads for a total of 19 samples. Table 1 summarizes the raw data from the sequencing of 14 samples analyzed. It is observed that the number of reads generated per sample ranged from 16,131,465 to 45,305 (Hi5 and Hi4 samples respectively), with an average percentage above 90% of bases with a phred score = 20. All analyzes performed are attached to this report with all contigs and genomic notations.

**Table 1:** Data of sequencing of *H. influenzae* strains.

Strain	Year	Site	R*eads number*	% Bases >= Q30	Sequencing average
AS1	2013	Blood	2,034,494	90.78	110.11
AS3	2012	Blood	5,652,459	92.23	305.92
AS4	2012	Blood	531,893	92.78	28.79
AS6	2014	Blood	3,131,435	92.18	169.48
AS11	2014	Blood	6,031,024	92.94	326.41
Hi1	2015	Oropharinx	4,603,214	92.48	249.13
Hi5	2015	Blood	16,131,465	93.6	873.06
Hi6	2015	Cerebrospinal fluid	9,851,427	94.38	533.17
Hi8	2015	Blood	9,353,479	92.92	506.22
Hi9	2015	Blood	15,383,481	94.66	832.57
Hi11	2015	Blood	12,344,279	94.29	668.09
Hi38	1997-1998	Blood	11,381,624	93.7	615.99
Hi45	1997-1998	Blood	10,710,597	93.43	579.67
HiP1	2019	Blood	3,245,950	89.26	175.68
HiX	2019	Blood	10,818,194	93.8	585.50

Still analyzing the properties and data of the sequenced genomes, the MLST (Table 2) analyses were performed in order to obtain a clonal analysis of the strains isolated in this study. Furthermore, strains isolated between 1997-1998 called ancestor strain (Hi38), strains isolated already in the years 2010, and strains isolated in 2019 (AS's and Hi's) were analyzed with a draft-whole genome. Regarding the MLST analysis of all strains, new alleles were found in our strains isolated in 2019 (*frdB* allele 232 and *recA* allele 191 of HiP1 and HiX, respectively). In addition, new sequences type for *H. influenzae* found in the Hi11, HiP1 and HiX strains (curated on the MLST website) were also determined.

**Table 2:** MLST genes and sequencing type of *H. influenzae* strains.

Strain	Adk	atpG	frdB	fucK	mdh	pgi	recA	ST
AS1	4	15	7	14	78	90	41	634
AS3	1	8	1	14	9	14	13	11
AS4	1	1	1	1	81	21	5	180
AS6	13	16	5	2	3	11	7	23
AS11	42	9	8	2	7	8	4	524
Hi1	14	51	16	48	29	2	31	556
Hi5	13	16	5	2	3	11	7	23
Hi6	13	16	5	2	3	11	7	23
Hi8	3	18	53	2	7	40	10	1813
Hi9	45	1	1	1	1	1	5	1417
Hi11	11	2	15	8	49	26	3	NA*
Hi38	4	17	4	1	2	9	6	4
Hi45	10	14	4	5	4	7	8	6
HiP1	52	11	232*	8	7	1	3	NA
HiX	13	16	5	2	3	11	191*	NA

⁎ New alleles submitted to https://pubmlst.org/hinfluenzae/.

**Fig. 1:**
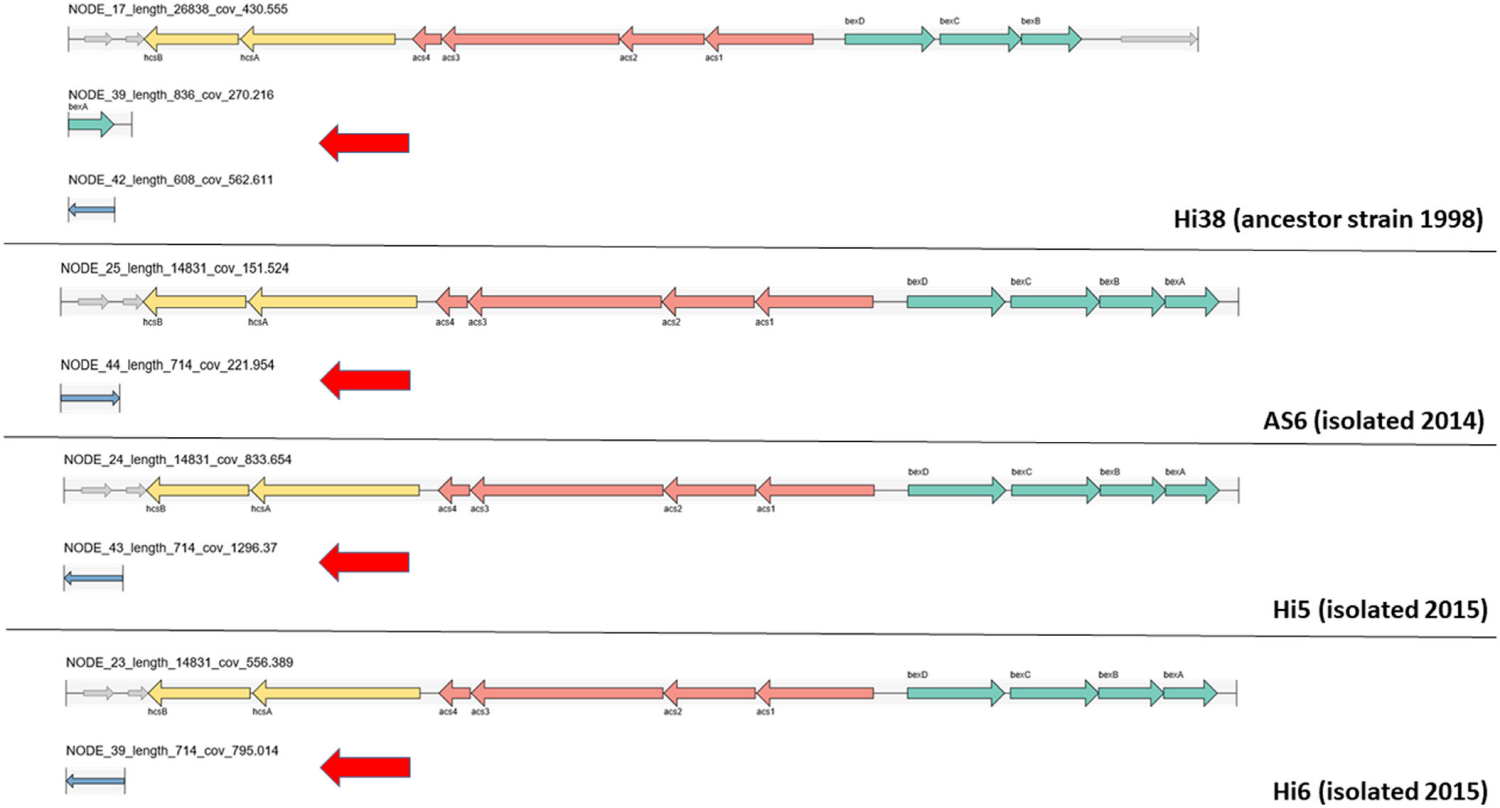
Schematic representations of recombination of the capsular operon from *H. influenza* serotype a comparing the old strain Hi38 (isolated in the 90s) and recent strains AS6, Hi5 and Hi6. The comparison of the strain Hi38 and the recent strains suggest a probable recombination process.

After assembling the genomic drafts, we observed the presence of NTHi and strains belonging to serotype a *H. influenzae* (strains AS6, Hi5 and Hi6) and to the same ST23. In the Fig. 1, the red arrows show the alterations in capsular operon in those strains. The correlation of the lineage considered elderly - Hi38 was found not to have the same clonal origin (Fig. 1).

## Discussion

Genome determination for studying *H. influenzae* strains associated with invasive diseases had been carried out by our group when Pereira *et al.* determined the whole-genome of *Haemophilus influenzae* that caused Brazilian purpuric fever in 2019.^5^ The expertise of genomic bioinformatics platforms made possible the analysis of invasive *H. influenzae* isolated in Clinical Hospital of Campinas State University in this study. This hospital health services covers all the metropolitan area of Campinas with around 3.2 million inhabitants and 20 cities.^17^^,^^18^

Thus, this analysis of bacterial populations is representative the Southwest Brazilian regions and the discovery of new variants of *H. influenzae* identified in this study is an important information for public health considering the identified new ST profile and the presence of serotype a *H. influenzae* in invasive diseases. Data presented in the supplementary material show several virulence genes detected in the strains analyzed in this study. Those virulence factors had been previously tested by our group as reported Pereira et al. 2021^19^ as expression of genes related with *H. influenzae* biotype aegyptius autotranporters. However, the supplementary analysis about other genes involved with the *Haemophilus* virulence could be a target for next investigations.

## Conflicts of interest

The authors declare no conflicts of interest.

## Appendix Supplementary materials

Image, application 1
