# Supplementary material for: Whole-genome analysis of haemophilus influenzae invasive strains isolated from Campinas state University hospital. An epidemiological approach 2012 - 2019 and ancestor strains
Source: Braz J Infect Dis. 2021 Dec 24;26(1):101667. doi: 10.1016/j.bjid.2021.101667 (PMC9387462; doi:10.1016/j.bjid.2021.101667)
Supplement: Supplementary file 1 [file mmc1.docx]

Table 1. Data to genome assembly and anotation

| Strain | Total Size (pb) | Size (%) * | Contigs | N50 (bp) | L50 | G+C (%) | CDS | RNA**t**s | RNA**r**s | Biosample** no. |
| --- | --- | --- | --- | --- | --- | --- | --- | --- | --- | --- |
| AS1 | 1,798,296 | 97 | 47 | 99,754 | 4 | 37,82 | 1.793 | 45 | 3 | SAMN15327300 |
| AS3 | 1,906,382 | 103 | 83 | 120,783 | 5 | 38,08 | 1.936 | 49 | 4 | SAMN15327301 |
| AS4 | 1,771,663 | 96 | 53 | 91,77 | 6 | 37,96 | 1.734 | 44 | 4 | SAMN15327302 |
| AS6 | 1,803,234 | 98 | 52 | 81,854 | 7 | 37,96 | 1.781 | 48 | 4 | SAMN15327303 |
| AS11 | 1,924,453 | 104 | 86 | 80,777 | 9 | 38,15 | 1.99 | 46 | 3 | SAMN15327304 |
| Hi1 | 1,801,552 | 98 | 52 | 79,341 | 6 | 38,01 | 1.775 | 46 | 10 | SAMN15327305 |
| Hi5 | 1,757,750 | 95 | 51 | 101,865 | 6 | 37,89 | 1.717 | 48 | 3 | SAMN15327306 |
| Hi6 | 1,749,971 | 95 | 48 | 101,767 | 5 | 37,87 | 1.711 | 47 | 4 | SAMN15327307 |
| Hi8 | 1,874,638 | 101 | 73 | 101,188 | 5 | 38,06 | 1.905 | 45 | 3 | SAMN15327308 |
| Hi9 | 1,818,015 | 98 | 59 | 89,177 | 6 | 37,99 | 1.787 | 49 | 4 | SAMN15327309 |
| Hi11 | 1,831,342 | 99 | 62 | 99,051 | 4 | 37,96 | 1.837 | 45 | 4 | SAMN15327310 |
| Hi38 | 1,800,739 | 97 | 49 | 100,915 | 5 | 38,06 | 1.77 | 44 | 4 | SAMN15327313 |
| Hi45 | 1,811,485 | 98 | 49 | 104,982 | 4 | 37,91 | 1.791 | 44 | 2 | SAMN15327314 |
| HiP1 | 1,872,921 | 101 | 72 | 59,566 | 7 | 37,98 | 1.892 | 46 | 4 | SAMN15327311 |
| HiX | 1,764,960 | 96 | 56 | 101,865 | 5 | 37,91 | 1.727 | 46 | 4 | SAMN15327312 |

*Size in relation to the reference strain and median of *H. influenzae* genomes in Genbank (1,847,700 pb).

** Biosamples belonged to Bioproject no PRJNA640662.
